# In vivo induction of membrane damage by β-amyloid peptide oligomers

**DOI:** 10.1186/s40478-018-0634-x

**Published:** 2018-11-29

**Authors:** Carl Julien, Colson Tomberlin, Christine M. Roberts, Aumbreen Akram, Gretchen H. Stein, Michael A. Silverman, Christopher D. Link

**Affiliations:** 1 Department of Integrative Physiology, University of Colorado at Boulder, Boulder, CO USA; 2 Department of Biological Sciences, and the Centre for Cell Biology, Development, and Disease, Simon Fraser University, Burnaby, British Columbia Canada; 3 Department of Molecular, Cellular, and Developmental Biology, University of Colorado, Boulder, Colorado 80309-0347 USA; 4 Centre de Recherche en Sciences Animales de Deschambault (CRSAD), Deschambault, Quebec, G0A 1S0 Canada; 5 Centre for Cell Biology, Development, and Disease), Simon Fraser University, British Columbia V5A 1S6, Burnaby, Canada

**Keywords:** Alzheimer’s disease, β-amyloid, Tau, *Caenorhabditis elegans*, Pore-forming toxin

## Abstract

Exposure to the β-amyloid peptide (Aβ) is toxic to neurons and other cell types, but the mechanism(s) involved are still unresolved. Synthetic Aβ oligomers can induce ion-permeable pores in synthetic membranes, but whether this ability to damage membranes plays a role in the ability of Aβ oligomers to induce tau hyperphosphorylation, or other disease-relevant pathological changes, is unclear. To examine the cellular responses to Aβ exposure independent of possible receptor interactions, we have developed an in vivo *C. elegans* model that allows us to visualize these cellular responses in living animals. We find that feeding *C. elegans E. coli* expressing human Aβ induces a membrane repair response similar to that induced by exposure to the CRY5B, a known pore-forming toxin produced by *B. thuringensis*. This repair response does not occur when *C. elegans* is exposed to an Aβ Gly37Leu variant, which we have previously shown to be incapable of inducing tau phosphorylation in hippocampal neurons. The repair response is also blocked by loss of calpain function, and is altered by loss-of-function mutations in the *C. elegans* orthologs of BIN1 and PICALM, well-established risk genes for late onset Alzheimer’s disease. To investigate the role of membrane repair on tau phosphorylation directly, we exposed hippocampal neurons to streptolysin O (SLO), a pore-forming toxin that induces a well-characterized membrane repair response. We find that SLO induces tau hyperphosphorylation, which is blocked by calpain inhibition. Finally, we use a novel biarsenical dye-tagging approach to show that the Gly37Leu substitution interferes with Aβ multimerization and thus the formation of potentially pore-forming oligomers. We propose that Aβ-induced tau hyperphosphorylation may be a downstream consequence of induction of a membrane repair process.

**Electronic supplementary material:**

The online version of this article (10.1186/s40478-018-0634-x) contains supplementary material, which is available to authorized users.

## Introduction

Alzheimer’s disease is characterized by the deposition in the brain of senile plaques, composed largely of the β-amyloid peptide (Aβ). The amyloid cascade hypothesis posits that in Alzheimer’s disease, accumulation of Aβ in the brain ultimately leads to changes in tau metabolism, which leads to the deposition of tau in neurofibrillary tangles (NFTs) that are the likely proximal cause of the neuronal loss observed in this disease [25]. While there is still significant controversy as to whether the amyloid cascade hypothesis explains Alzheimer’s pathology [28, 39, 72], there is extensive evidence (in vitro and in vivo) that exposure of neurons to Aβ can lead to tau hyperphosphorylation, a potential driver of tau deposition [23, 51, 87]. Multiple tau kinases have been identified (reviewed in [57]), as have pathways by which these kinases could be activated to increase tau phosphorylation [15, 55, 89]. CDK5 and GSK3β have emerged as strong candidates for disease-relevant tau kinases [27, 50], and activation of both these kinases can occur downstream of calpain activation. Calpains are calcium-dependent proteases that can cleave P35 to produce P25, a strong activator of CDK5 [81] or directly truncate GSK3β, leading to its activation [34]. Many studies have also demonstrated that exposure of neurons to Aβ can lead to calcium influx [30, 77, 78] and thus there exists a well-supported molecular mechanism explaining how Aβ exposure can lead to tau hyperphosphorylation. Less clear is the biological rationale for Aβ-induced tau phosphorylation. Is this a selected function of Aβ, an indirect consequence of an evolved interaction of Aβ with cells, or an essentially random interaction with potentially deleterious outcomes?

Addressing “why” Aβ induces tau phosphorylation is complicated by uncertainty as to what, if any, selected function the Aβ peptide has. Similarly unresolved is how extracellular Aβ induces intracellular increases in calcium. Aβ toxicity in numerous models can be attenuated by blocking the NMDA-type glutamate receptor [5, 11, 24], suggesting that this calcium channel could be responsible for Aβ-dependent calcium influx. Similarly, the prion protein has been claimed to be a receptor for Aβ oligomers [7, 44, 74] and to moderate Aβ-dependent calcium influx [67]. Other studies have implicated AMPA glutamate receptors [2, 73, 88] or L-type voltage gated calcium channels [4] in cytoplasmic calcium increases resulting from Aβ exposure. While multiple calcium channels could be involved in Aβ-dependent calcium influx, other studies suggest that Aβ oligomers could act independently of endogenous calcium channels by directly forming a calcium-permeable pore. It has long been known that synthetic Aβ can form ion-permeable channels in synthetic membranes [6], a finding that has often been replicated [29, 41, 71]. Although ring-like structures of Aβ oligomers have been visualized by atomic force microscopy in synthetic membranes [46], Aβ membrane pores in pathologically-relevant cells have not been directly visualized or assayed.

One approach to determining if Aβ pores are relevant to Aβ neurotoxicity is to identify substitutions in the Aβ sequence that block pore formation in synthetic membranes, and then determine if these substitutions alter Aβ toxicity. Kim et al. [41] noted a sequence motif (Gly-XXX-Gly-XXX-Gly) present in both the C terminus of Aβ and the transmembrane domains of bacterial channel proteins, and they proposed that this motif (the “glycine zipper”) could facilitate α-helical interactions driving Aβ oligomer assembly in membranes and subsequent pore formation. These researchers demonstrated that leucine substitutions at these glycine residues (particularly Gly^37^Leu) prevented synthetic Aβ 1–42 from forming ion channels in synthetic membranes and also significantly reduced Aβ toxicity in Neuro 2A cells. Fonte et al. [20] extended these results and demonstrated that the Gly^37^Leu substitution both reduced Aβ toxicity in an in vivo transgenic *C. elegans* model that expresses human Aβ and prevented synthetic Aβ oligomers from inducing tau hyperphosphorylation in hippocampal neurons. Peters et al. [68] subsequently demonstrated that a pentapeptide derived from the glycine zipper sequence (GLMVG) inhibited Aβ synaptotoxicity. These observations are consistent with pore formation underlying Aβ neurotoxicity, but they do not directly demonstrate pore formation, and they cannot exclude other interpretations, such as the possibility that the glycine zipper substitutions interfere with interactions with specific cell surface receptors.

**Fig. 1:**
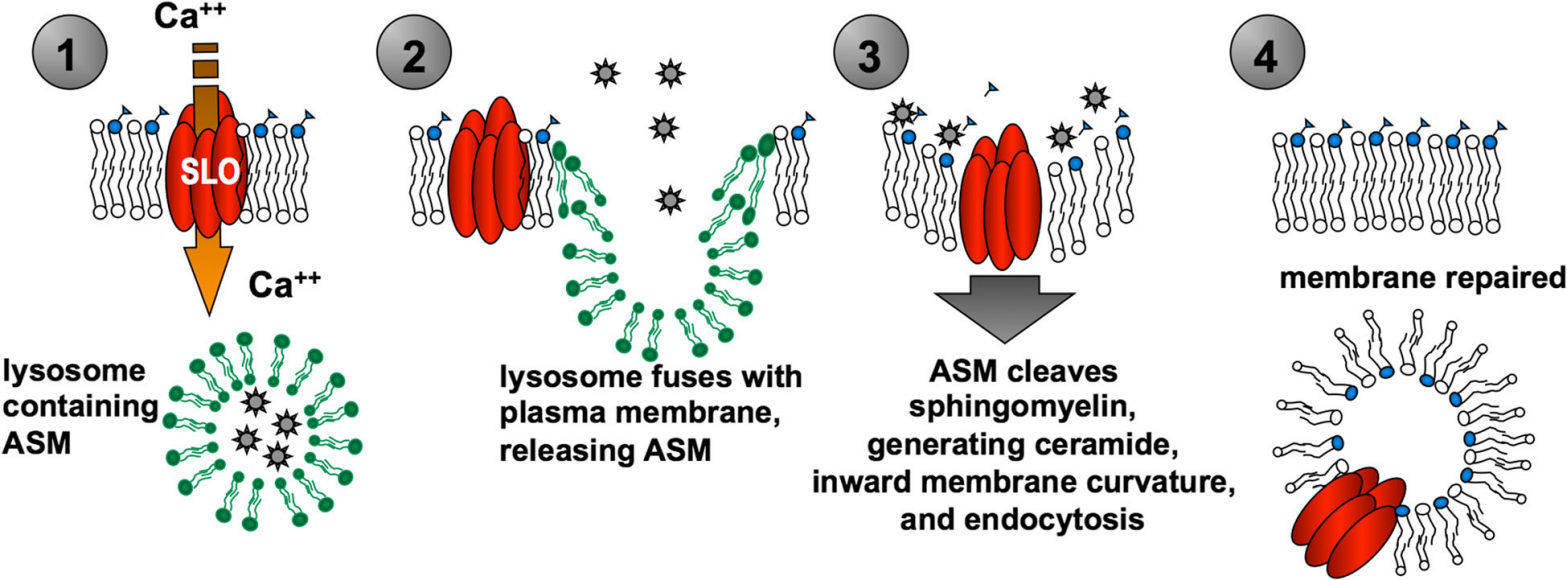
Membrane repair model. This model is based on the mechanism for repair of plasma membrane (PM) pores created in mammalian cells by exposure to the bacterial pore-forming toxin SLO [32]. ASM is acidic spingomyelinase that is stored in lysosomes, which fuse to the PM in response to an influx of Ca2+ through the pore created by SLO (Step 1), thereby releasing ASM at the cell surface (Step 2). The localized release of ASM cleaves off the phosphoryl head group of sphingomyelinase in the vicinity of the pore to generate ceramide in that area (Step 3). Consequently, the PM around the pore undergoes inward curvature and endocytosis such that the pore is removed from the PM (Step 4)

An alternative approach to inferring pore formation by Aβ is to look for its ability to induce a known biological response to exogenously induced pores, rather than attempting to image the pores themselves. Andrews and colleagues have defined a membrane repair pathway that occurs in response to exposure to streptolysin O, (SLO), a well-characterized bacterial pore-forming toxin [32]. As outlined in Fig. 1, this multi-step repair pathway involves: 1) calcium influx through the SLO pore, 2) fusion of local lysosomes with the plasma membrane, releasing lysosomal contents, 3) cleavage of nearby sphingolipids in the outer leaflet of the plasma membrane by lysosomally-derived acid sphingomyelinase, thereby inducing localized inward membrane curvature, and 4) removal of the pore-containing plasma membrane by endocytosis. While different membrane repair mechanisms are apparently employed for different classes of pore-forming toxins [19], if Aβ-induced calcium influx results from an SLO-analogous pore, three strong predictions can be made: 1) Aβ exposure will induce sphingomyelinase-dependent endocytosis, 2) non-toxic Aβ variants (e,g., Aβ_42_ Gly^37^Leu) will be incapable of inducing membrane repair because they cannot appropriately oligomerize to form membrane pores, and 3) exposure to pore-forming toxins will mimic the effects of Aβ oligomers, specifically the hyperphosphorylation of tau. Here we test these predictions using a novel *C. elegans* model and primary cultures of rat hippocampal neurons.

Transgenic *C. elegans* strains have been constructed that express human Aβ_42_ [16–18, 47, 82], and these strains have a variety of phenotypes, depending on where the transgene is expressed. A confound of these models is that the detectable Aβ is intracellular when assayed by immunohistochemistry, so the degree of outside-in Aβ toxicity (i.e., extracellular Aβ affecting neighboring cells) is unclear. To circumvent this limitation, we have developed a “feeding” model, where *C. elegans* is fed *E. coli* engineered to secrete human Aβ, and the cellular effects of this exogenous peptide are assayed in intestinal cells. One rationale for this model is that the *C. elegans* intestine does not express candidate Aβ receptors (e.g., prion protein, NMDA glutamate receptors, α7nAChR, etc.), and thus any physiological response to Aβ is unlikely to be receptor-mediated. The transparency of *C. elegans* and the existence of relevant transgenic reporter strains allows the effects of Aβ exposure to be followed in live intact animals. Using this model, we show that exposure to wild type Aβ_42_ (but not Aβ_42_ Gly^37^Leu) induces acid-sphingomyelinase-dependent endocytosis that parallels the response to a known pore-forming toxin, CRY5B. We find that this response to Aβ is calpain-dependent and is altered by loss-of-function mutations in the *C. elegans* orthologs of BIN1 and PICALM, two Alzheimer risk genes identified in genome-wide association studies [61, 84]. In hippocampal cultures, we show that the SLO pore-forming toxin induces calpain-dependent tau phosphorylation in primary neurons. Furthermore, exogenous sphingomyelinase itself can induce increased tau phosphorylation in these neurons. Finally, we use a novel tagging method to show that the Gly^37^Leu substitution does inhibit Aβ multimerization in a cellular context, thereby rationalizing why this Aβ variant is incapable of inducing membrane repair or tau phosphorylation. Taken together, these results support the view that tau hyperphosphorylation may be a downstream consequence of a membrane repair process, and that exogenous Aβ can induce membrane repair because of its ability to oligomerize into membrane pores.

## Materials and methods

### *C. elegans* strains and maintenance

*C. elegans* strains were generally maintained at 20 °C using standard methods [8]. Strains containing the *rynEx60* transgene were passaged at 25 °C to select for retention of this *pha-1* rescuing extrachromosomal transgene.

*C. elegans* strains used in this study.

### Construction of *E. coli* feeding strains

To engineer *E. coli* strains capable of secreting human Aβ, plasmids were constructed by inserting the wild type or Gly^37^Leu Aβ sequence between the NcoI and SacI sites of the arabinose-inducible pBAD gIII vector (Invitrogen), yielding pCL241 and pCL242, respectively. These plasmids were transformed into *E. coli* strain LMG194, allowing tight control of arabinose induction.

### Feeding protocol and endosome scoring in *C. elegans*

Overnight cultures of *E. coli* strains were diluted 1:100 in Luria broth (+ 100 μg/mL ampicillin) and grown for 2 h in an orbital shaker at 37 °C. Twenty percent arabinose was added to final concentration of 0.2%, and the cultures were grown for another hour. These *E. coli* cultures were then spread on NGM plates (nematode growth media, [8]) containing 0.2% arabinose and 100 μg/mL ampicillin and used the next day. L4 worms were transferred onto the fresh plates and allowed to feed for 4 h at 25 °C. Worms were imaged with a Zeiss Axiophot microscope (40X objective) and endosomes were counted in the anterior 50 μm of the intestine.

### Hippocampal neuronal culture

Different protocols were used for the preparation of hippocampal neurons in the Stein and Silverman labs. Results obtained in replicate experiments using the two different protocols were highly reproducible.

(Stein lab) Hippocampal neurons were isolated from rat E18 hippocampus (BrainBits, LLC) according to the supplier’s protocol. The neurons were plated at 10–20,000 cells/cm^2^ on Lab-Tek CC2 4-well chamber slides coated with poly-D-lysine (Sigma P0899) and grown at 37 ^o^ C. The medium, Neurobasal + 2% B27 and 0.5 mM GlutaMax (Invitrogen), was supplemented with 25 mM glutamate for the first 4 days; thereafter the neurons were fed twice a week with medium lacking glutamate and were used for experiments after 9–16 days in vitro (DIV).

(Silverman lab) Primary hippocampal neuronal cultures from E18 embryonic rats (Charles River, USA) of either sex were prepared as described by Kaech and Banker [35] and kept in PNGM primary neuron growth media (Lonza, Basel, Switzerland). The glial feeder layer was derived from murine neural stem cells as described by [59]. All experiments with animals were approved by and followed the guidelines set out by the Simon Fraser University Animal Care Committee, Protocol 943-B05.

### SLO activation and treatment of cells

SLO (Aalto Bio Reagents), a thiol-activated pore-forming toxin that loses activity upon storage at − 70^0^ C, was reactivated before each experiment by incubating an aliquot of 3 mg/ml SLO (in Tris-buffered saline, pH 8.5) with an equal volume of 20 mM DTT for 10–15 min at room temperature. Activated SLO and vehicle were diluted with medium and added directly to the cells as 6X concentrates, or half the old medium on the cells was removed and replaced by a 2X concentration of SLO or its vehicle control. The toxicity of each aliquot of activated SLO was evaluated by exposing rat insulinoma cells (RIN5F in RPMI 1640 with 10% Hyclone FBS) to 0–2000 ng/ml SLO for 2 h and measuring viability by the reduction of MTT (Sigma M5655). The results indicated that SLO had little or no gross toxicity at < 200 ng/ml and killed half the cells at 600–800 ng/ml. We treated neurons with 50–100 ng/ml SLO for 1–2 h.

### Treatment with calpain inhibitor (PD 150606)

PD150606 (Sigma D5946) was dissolved in DMSO, aliquoted and stored at -20^o^ C. It was added to cells at a final concentration of 30 μg.

### Treatment with sphingomyelinase

Ten units of lyophilized *B. cereus* sphingomyelinase (SMase, Sigma S7651) were dissolved in 200 μl of cold 50% glycerol in sterile Ca^2+^/Mg^2+^ free PBS, yielding 50 mU/μl. Because SMase loses activity even when stored at − 70^0^ C, it was best used fresh. Neurons were treated with vehicle control and SMase diluted to 2.5 mU/ml in Neurobasal/B27 for 2 h.

### Antibodies for immunostaining neurons or *C. elegans*

Primary antibodies used to stain neurons were mouse monoclonal PHF1 to p-Ser396/p-Ser404 tau (kind gift from Dr. Peter Davies), mouse monoclonal AT8 to p-Ser202/p-Thr205 tau (Pierce, Thermo) and rabbit polyclonal K9JA to total tau (Dako A0024). Secondary antibodies (goat anti-rabbit IgG Alexa Fluor 488, goat anti-mouse Alexa Fluor 594 and donkey anti-mouse IgG Alexa Fluor 555) and Prolong Gold Antifade mounting medium were from Invitrogen. The biarsenical dye used was from the TC-FlAsH™ II In-Cell Tetracysteine Tag Detection Kit (Molecular Probes, Eugene, OR). The anti-RME-1 antibody was obtained from the Developmental Studies Hybridoma Bank, and anti-Aβ mouse monoclonal 6E10 was purchased from BioLegend.

### Immunocytochemistry

Neurons grown on chamber slides (Stein lab) were fixed for 15 min in 4% p-formaldehyde/4% sucrose, permeabilized 7 min in 0.25% Triton X-100, and blocked 1–2 h with 5% goat or sheep serum (all solutions in PBS). Slides were stained with primary antibodies (K9JA at 1:500, and AT8 or PHF1 at 1:50), secondary antibodies at 1:500, and mounted with ProLong Gold AntiFade. Images were obtained by using a defined scanning pattern to view the total tau fluorescence in sequential fields in each well, capturing images of fields with an average number of cells (typically 5–20 cells per field) using a 10X or 20X objective on a Zeiss Axioskop epifluorescence microscope equipped with 3i Slidebook image analysis software. Identical conditions and exposure times were used to capture an average of 18.5 fields of cells for each treatment presented in our figures, and the ratio of p-tau to t-tau was determined for each image. The mean for each treatment was calculated and compared to its control, and Student’s t-test was used to determine if the difference of the means was significant. Similarly, neurons grown on coverslips (Silverman lab) were fixed in 4% paraformaldehyde for 15 min, blocked in 0.5% fish skin gelatin and 0.1%Triton X-100 in PBS, and immunostained with K9JA and AT8 or PHF1. In this case, to quantify tau phosphorylation, histograms were generated using ImageJ from the fluorescence intensity of each pixel across several images, and the average intensity was calculated [20]. Appropriate thresholds were applied to eliminate background signal before histogram analysis, and 17 images per experimental condition were analyzed from at least three independent neuronal cultures.

### Preparation of Aβ oligomers

Aβ oligomers were prepared from synthetic Aβ (BioSynthesis) using the “ADDL” preparation originally described by Lambert et al. [43], and subsequently used by us to characterize wild type and Gly^37^Leu oligomers [20]. Briefly, peptides were solubilized in hexafluoroisopropanol (HFIP) and desiccated in microfuge tubes, then dissolved in fresh, anhydrous DMSO (Sigma Hybri-Max D-2650) to make a ~ 5 mM solution. This solution was then diluted to ~ 100 μM with cold F12 media without phenol red (Biosource) and aged 24 h at 4 °C. The samples were centrifuged at 14,000 g for 10 min at 4 °C to remove any insoluble material, and the supernatants stored at 4 °C.

### Immunoblotting

Neurons were treated with vehicle, SMase, SLO, and PD 150606 as described above and lysed in RIPA buffer containing Complete Protease Inhibitor Cocktail (Roche) and Halt Phosphatase Inhibitor Cocktail (Thermo Fisher). Samples (10 μg) were resolved on 10% SDS–polyacrylamide gels and transferred to polyvinylidene fluoride (PVDF) membranes. Membranes were incubated with the following primary antibodies overnight at 4 °C: PHF-1 (1:1000), AT8 (1:250), K9JA (1:2000), and anti–tubulin (1:1000). The membranes were imaged using Fujifilm LAS4000 Luminescent Imager. Densitometric scanning and quantitative analysis were carried out using ImageJ.

For assaying Aβ expression in engineered *E. coli* strains, bacterial cultures were spun down in a tabletop centrifuge (3 min at 10,000 rpm), and pellets were frozen at − 80 °C until use. Pellets were solubilized in RIPA buffer supplemented with AEBSF (Sigma P2714) and quantitated using Pierce BCA Protein Assay (Thermo Fisher 23,225). Protein samples were boiled in sample buffer (4% BME in NuPAGE LDS sample buffer (Invitrogen NP0007) and run at 180 V on NuPAGE 4–12%Bis-Tris Gels (Invitrogen, NP0321) using MES SDS Running Buffer (Invitrogen NP0002). Gels were transferred to 0.45 μm supported nitrocellulose (GE Osmonics WP4HY00010) using 20% methanol, 39 mM glycine, 48 mM Tris base at 21 V for 108 min. Prestained Rainbow size markers (Amersham Biosciences RPN755) were used to size bands. Blots were visualized by Ponceau stain, boiled for 3 min in PBS, blocked in TBS-Tween + 5% milk (100 mM Tris7.5, 150 mM NaCl, 0.1% Tween-20) and probed with Aβ antibody 6E10 (BioLegend 803,002) at a 1:1000 dilution. Secondary HRP-conjugated antibody (Sigma A5906 mouse) was used, and the blot was developed in ECL Plus (Amersham RPN2132).

### Treating hippocampal neurons with Cys-tagged Aβ peptides

Rat hippocampal neurons (cultured in the Stein lab) were treated with wild-type and Cys-tagged Aβ peptides (synthesized by Bio Synthesis) and exposed to the FlAsH dye according to manufacturer’s instructions with modifications [TC-FlAsH™ II In-Cell Tetracysteine Tag Detection Kit (Molecular Probes)]. Briefly, rat hippocampal neurons were treated to 10 μM EDTA to suppress background fluorescence for 10 min, and were then exposed to 2.5 μM Cys-tagged Aβ peptides or wild-type Aβ peptides for 1 h at 37 °C. FlAsH dye was then applied to neurons at 1:800 for 30 min. BAL wash buffer was used to remove the excess of the FlAsH dye and was replaced by warm HBSS after 15 min. Neurons were then immediately fixed and prepared for immunohistochemistry.

### Microscopy

A Zeiss Axiophot microscope equipped with digital deconvolution optics and a Nikon Structured Illumination Super-resolution (Light Microscopy Core Facility in Department of Molecular, Cellular and Developmental Biology, University of Colorado Boulder) were used for imaging *C. elegans* and rat hippocampal neurons. Images generated in the Silverman lab were acquired using a Leica DMI6000B inverted epifluorescence microscope using a 63 X 1.4 N.A. oil-immersion objective equipped with a cooled CCD camera controlled by MetaMorph (Molecular Devices).

### Statistical analysis

A one-tailed paired two sample for mean t-test, was used to determine significance between pair wise comparisons of control and experimental conditions. Log rank statistics were used to analyze survival curves in the CRY5B exposure experiments (Fig. 5b). Significant differences between the treatments were analyzed by t-tests with equal or unequal variance at a 95% confidence interval. The experiments were carried out in three different cultures and at least 60–70 cells were analyzed per condition. For the blots, cell lysates were collected from at least 3 cultures.

## Results

### Aβ induces a membrane repair process in *C. elegans*

**Fig. 2:**
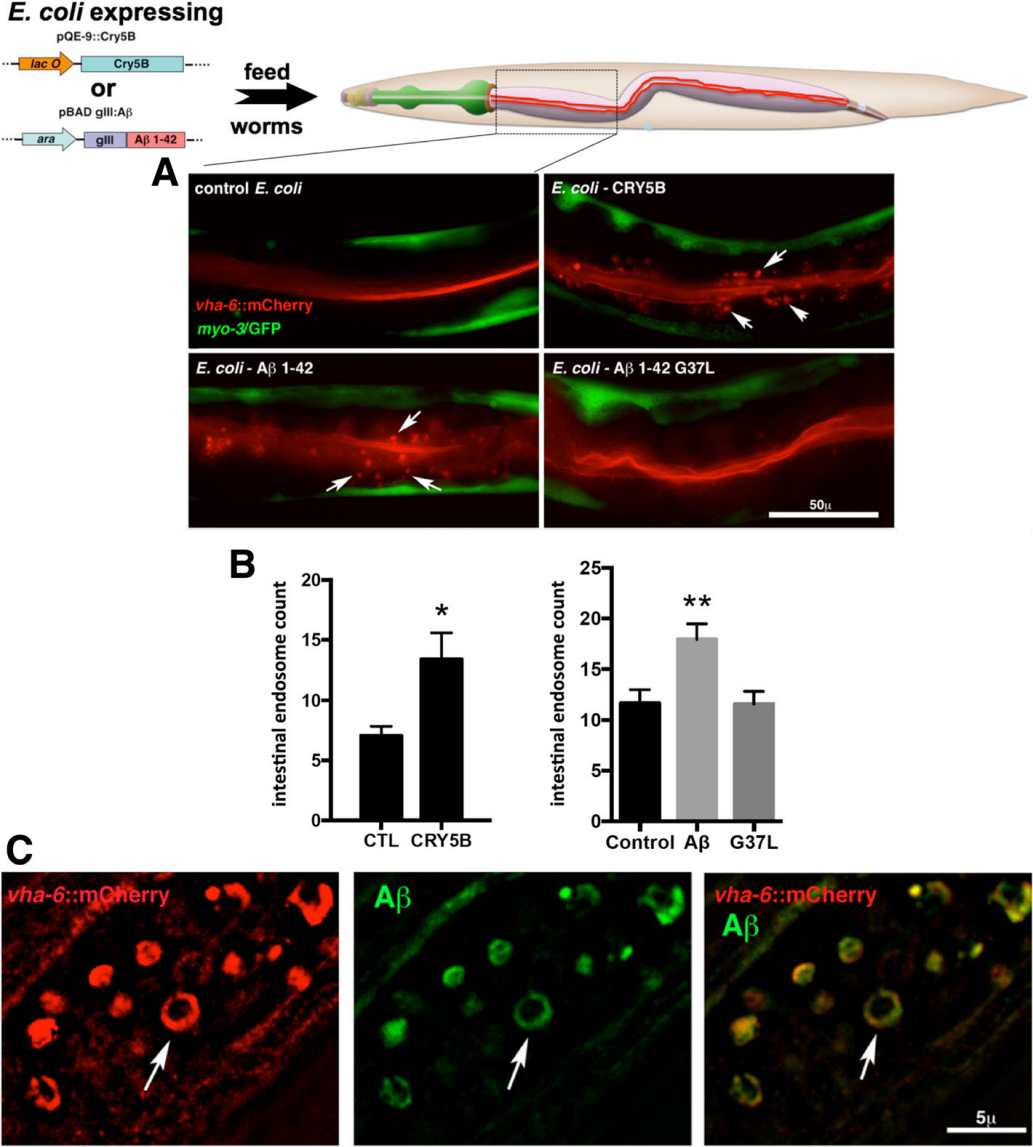
Induction of intestinal endosomes in *C. elegans* fed *E. coli* that express human Aβ peptide. **a** Live images of *C. elegans* reporter strain KWN117 (*vha-6*::mCherry) fed *E. coli* expressing either vector control, CRY5B toxin, wild type Aβ_1–42_, or Aβ_1–42_ Gly^37^Leu. Note that both CRY5B and wild type Aβ_1–42_ induce intestinal endosomes (arrows) containing the *vha-6*::mCherry reporter usually associated with the lumenal membrane of the intestine. This induction does not occur in worms fed *E. coli* expressing the non-pore-forming Aβ_1–42_ Gly^37^Leu variant. **b** Quantification of endosome induction. **c** Super resolution image of intestinal endosomes induced in strain KWN117 by wild type Aβ_1–42_. The treated worm was subsequently fixed, permeabilized, and probed using anti-Aβ antibody 6E10. Note that the Aβ staining co-localizes with the membrane-associated *vha-6*::mCherry reporter (arrow), not the endosomal lumen. *p < 0.05 and **p < 0.01 when compared with the vehicle control

To assay the ability of human Aβ to induce membrane repair in an in vivo model, we took advantage of previous work by Aroian and colleagues, who studied the effects of the CRY5B toxin on *C. elegans* [31, 38, 85]. The CRY5B protein is a member of the “Bt crystal” collection of pore-forming toxins secreted by *Bacillus thuringiensis*. To visualize the effects of CRY5B on *C. elegans* intestinal cells, these researchers used a transgenic reporter strain that expresses a fluorescent fusion protein, specifically localized to the lumen of the intestine, to monitor endocytosis following exposure to an *E. coli* strain engineered to express CRY5B. We have replicated these experiments (Fig. 2a, right top panel), and as previously reported [52], exposure to CRY5B results in a significant induction of endocytosis, a component of the intestinal membrane repair process. In parallel, we engineered *E. coli* to secrete Aβ 1–42, using the arabinose-inducible vector pBAD gIII, and exposed reporter strain KWN117 (*vha-6*::mCherry) to those induced *E. coli* cultures. As shown in Fig. 2a, this resulted in an induction of endocytosis similar to that observed in the CRY5B-exposed worms. The increased intestinal vesicles observed in these experiments could be labeled with endosomal marker RAB-5::GFP or immunostained with antibodies against the endosomal RME-1 protein, demonstrating that these vesicles are indeed endosomes (Additional file 1: Figure S1 A, B). Importantly, this induction of endocytosis was not observed in KWN117 worms exposed to an *E. coli* strain expressing the Gly^37^Leu variant of Aβ, which we have previously shown to be non-toxic in mammalian neurons [20]. (The engineered *E. coli* strain expressing the Aβ_42_ Gly^37^Leu variant actually produces higher levels of Aβ peptide than the Aβ_42_ wild type strain when assayed by immunoblot, Additional file 1: Figure S1C).

**Fig. 3:**
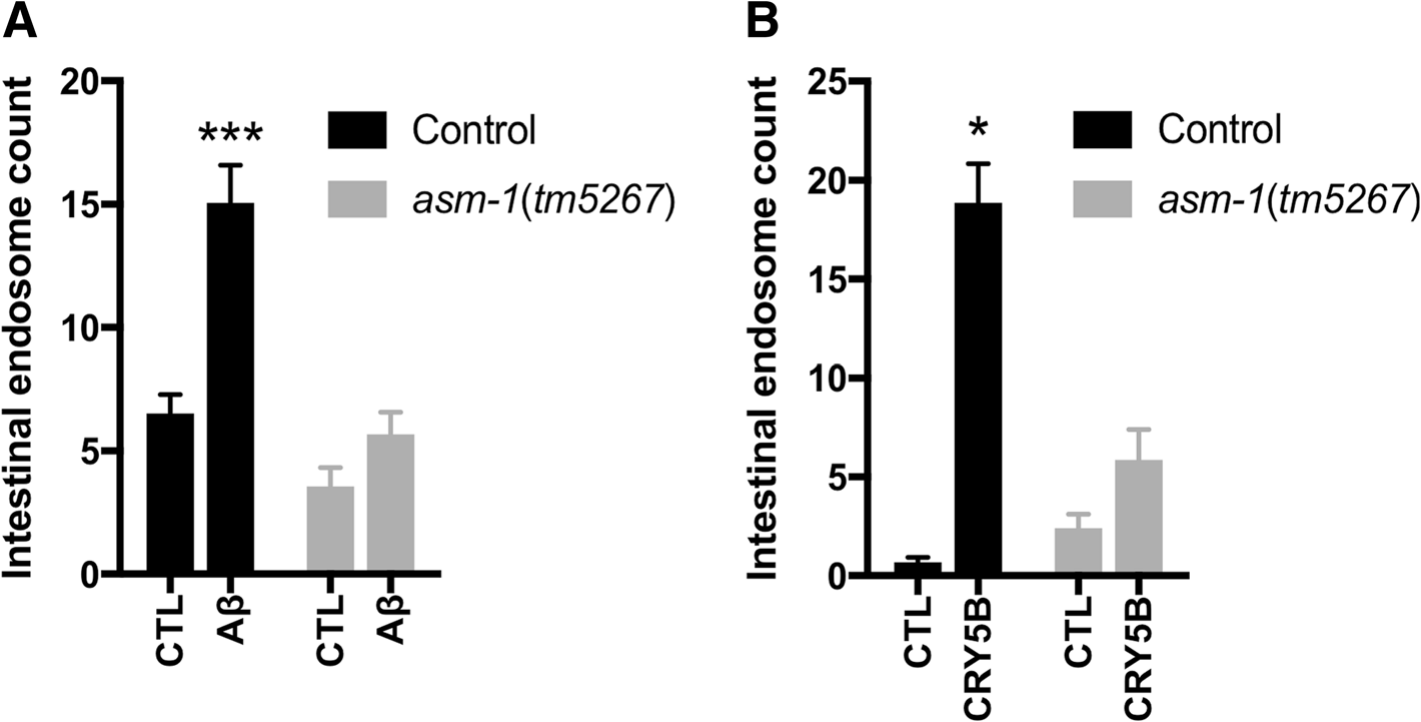
Induction of intestinal endosomes is blocked by a mutation in the acid sphingomyelinase gene *asm-1*. **a** Introduction of the *asm-1*(tm5267) deletion allele into reporter strain KWN117 blocks the ability of *E. coli* expressing wild type Aβ_1–42_ to induce intestinal endosomes. **b** The *asm-1*(tm5267) mutation also blocks endosome induction by the CRY5B toxin. *p < 0.05 and ***p < 0.001 when compared with the vehicle control

If the intestinal endosomes induced by exposure to Aβ-expressing *E. coli* were in fact a result of a membrane repair process similar to that proposed by Andrews and colleagues (Fig. 1), our expectation was that these endosomes would contain membrane-associated Aβ that had been endocytosed from the luminal plasma membrane. We therefore fixed and permeabilized KWN117 reporter worms fed *E. coli* expressing Aβ, then immunostained with an Aβ-specific antibody (mAB 6E10). Super-resolution microscopy revealed cytoplasmic rings in intestinal cells that contained both the VHA-6::mCherry luminal marker and Aβ (Fig. 2b). We interpret these rings as cross-sections of endosomal vesicles containing both membrane-associated Aβ and VHA-6::mCherry, consistent with the pore-removal-by-endocytosis model proposed by Andrews and colleagues. A critical component of this model is the initial exocytosis of acid sphingomyelinase, a lysosomal enzyme that when released extracellularly can cleave sphingomyelin in the outer leaflet of the plasma membrane lipid bilayer. This conversion of sphingomyelin to ceramide can lead to inward membrane curvature, which is thought to promote local endocytosis (Fig. 1). To test further whether the *C. elegans* response to Aβ exposure paralleled the Andrews model, we repeated the feeding experiments using a derivative of the *vha-6*::mCherry reporter strain that contained the *asm-1 (tm5627)* mutation, a deletion allele of a sphingomyelinase gene expressed in the intestine. We observed that loss of *asm-1* blocked endosome induction by feeding with CRY5B or Aβ-expressing *E. coli* (Fig. 3), again supporting the Andrews model.

**Fig. 4:**
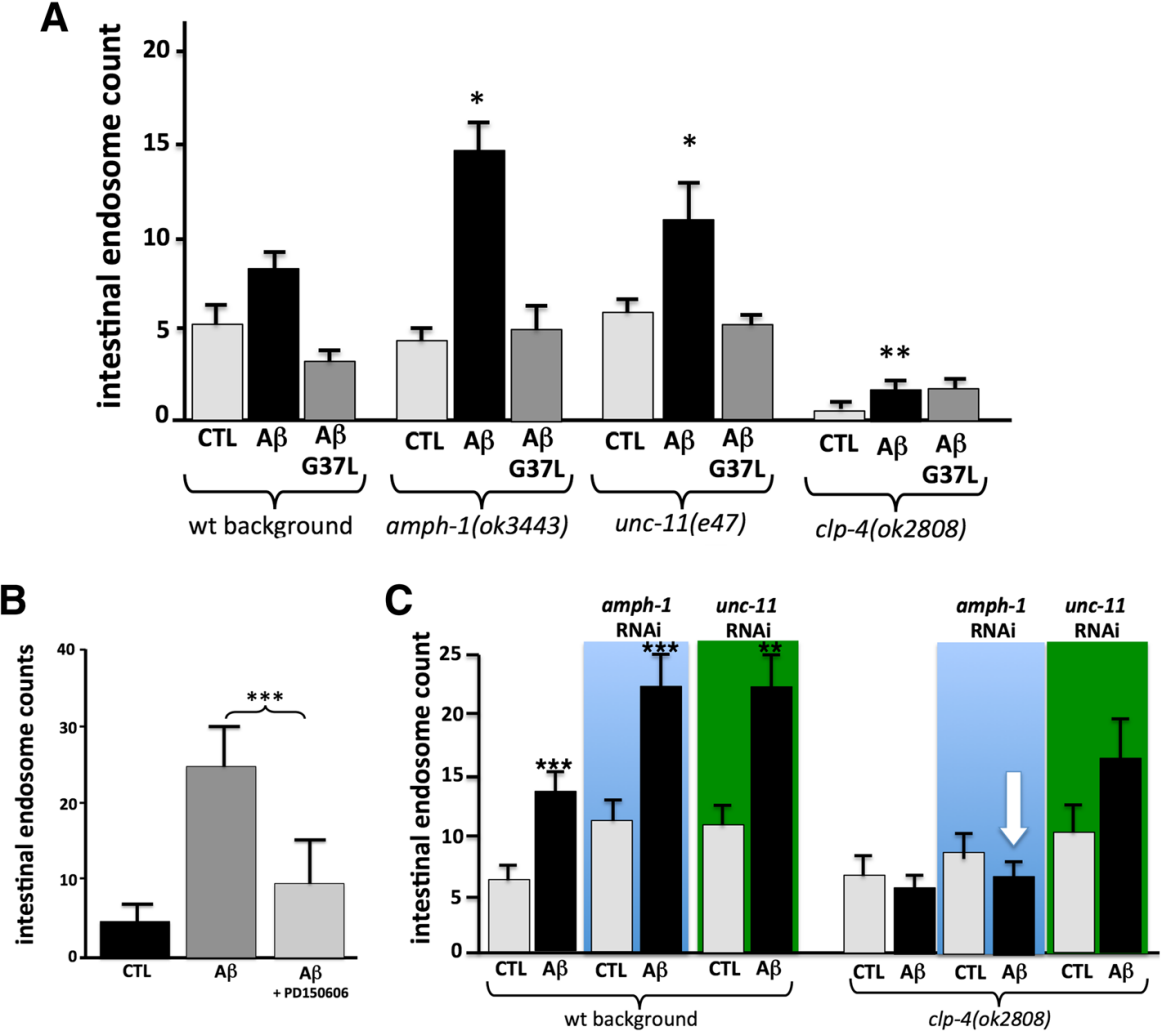
Effects of *amph-1*, *unc-11* and *clp-4* mutations on Aβ-induced intestinal endosomes. **a** Strains containing the *vha-6*::mCherry reporter and deletion mutations in *amph-1*, *unc-11*, or *clp-4* were fed *E. coli* expressing wild type Aβ_1–42_. Note that the *amph-1* and *unc-11* mutations increase, while the *clp-4* mutation decreases, the accumulation of intestinal endosomes. **b** Treatment with the calpain inhibitor PD150606 replicates the ability of the *clp-4* mutation to block Aβ-induced increases in intestinal endosomes. **c** RNAi treatments indicate the *clp-4* deletion mutation is epistatic to the effects of *amph-1* and *unc-11* knockdown. *p < 0.05, **p < 0.01 and ***p < 0.001 when compared with the vehicle control

To explore the possibility that some genes implicated in Alzheimer’s disease might also be involved in the Aβ-induced membrane repair process, we examined the effects of loss-of-function mutations in *amph-1*, *unc-11*, and *clp-4* on Aβ-induced endocytosis. *Amph-1* is the *C. elegans* ortholog of BIN1, a gene associated with Alzheimer’s risk in multiple genome-wide association studies [10, 75]. BIN1 is a member of the Bin1/amphiphysin/RVS167 (BAR) family of proteins that are involved in diverse cellular processes, including endocytosis. *Unc-11* is orthologous to PICALM, another well-established late onset Alzheimer’s disease (LOAD) risk gene known to play a role in clathrin-mediated endocytosis [40]. *clp-4* encodes one of nine *C. elegans* calpains, and we have previously noted that this specific calpain is upregulated both in a transgenic *C. elegans* model of Aβ toxicity [26] and in worms exposed to CRY5B [31]. The CLP-4 calpain also activates the *C. elegans* CDK5 ortholog via cleavage of p35 to p25 [58], and thus has functions analogous to human calpains 1/2, which are required for Aβ-induced tau phosphorylation via p25 activation of CDK5 [50, 81]. As shown in Fig. 4a, introduction of either an *amph-1* deletion allele or an *unc-11* null allele (*e47*) into the *vha-6*::mCherry reporter background led to a significant increase in endosomes induced by Aβ feeding. (Similar results were observed when these genes were knocked down by RNAi, or if CRY5B exposure was used to induce endocytosis, Additional file 1: Figure S2 A, B.) In contrast, introduction of a *clp-4* deletion allele blocked the increase of endosomes induced by Aβ feeding. The effects of the *clp-4* deletion can also be mimicked by pre-treating the reporter strain with a specific calpain inhibitor, PD150606 (Fig. 4b).

**Table:** 

Strain	Genotype
KWN117	*pha-1*(e2123) III; *him-5*(e1490) V; ***rnyEX60*** (*vha-6*::mCherry, P*myo-3*:: GFP, *pha-1* rescuing fragment)
GK280	*unc-119*(ed3) III; ***dkIs166***(P*opt-2*::GFP::*pgp-1*; *unc-119* rescuing fragment)
CL6651	*asm-1*(tm5267) II; *pha-1*(e2123) III; *him-5*(e1490) V; ***rnyEX60***
CL6626	*pha-1*(e2123) III(?); *amph-1*(ok3443); *him-5*(e1490) V (?); ***rnyEX60***
CL6605	*unc-11*(e47) I; *pha-1*(e2123) III (?); *him-5*(e1490) V (?); ***rnyEX60***
CL6623	*clp-4*(ok2808) III; *him-5*(e1490) V (?); ***rnyEX60***
RT327	*unc-119*(ed3) III; ***pwIS72***[P*vha-6*::GFP::*rab-5*, *unc-119* rescuing fragment)

To examine the epistatic relationship between these genes, we repeated the Aβ feeding assay after knocking down *amph-1* or *unc-11* by RNAi in the reporter strain, with and without the *clp-4* mutation. We found that the increased endocytosis induced by *amph-1* RNAi was completely blocked by the *clp-4* loss-of-function mutation, and the increased endocytosis induced by *unc-11* RNAi was somewhat reduced (Fig. 4c). Thus, *clp-4* is fully epistatic to *amph-1* and may moderate *unc-11* in the membrane repair process.

**Fig. 5:**
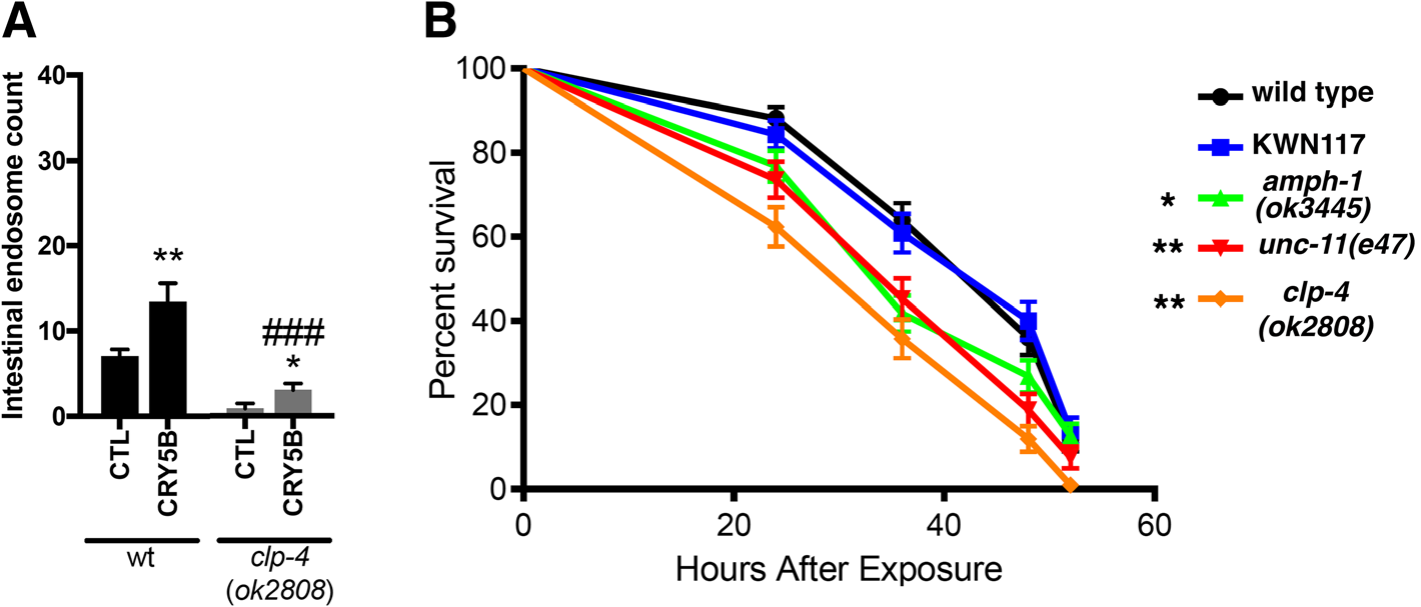
*clp-4* deletion blocks endosome induction by CRY5B and sensitizes worms to CRY5B toxicity. **a** Induction of endosomes induced by CRY5B is blocked by the *clp-4*(ok2808) deletion mutation. *p < 0.05 when compared with the vehicle control. ###*p* < 0.001 when compared with the wt background. **b** Survival curves of *C. elegans* strains exposed to *E. coli* expressing CRY5B. Note that all three mutations that alter endosome accumulation also sensitize worms to CRY5B toxicity. *p < 0.05, **p < 0.01 compared to wild type control. NS = not significant

If *clp-4* plays a general upstream role in membrane repair, loss of *clp-4* should also block induction of endocytosis by CRY5B, and should be deleterious when *C. elegans* is challenged by this pore-forming toxin. As observed for Aβ feeding, introduction of the *clp-4* deletion allele into the *vha-6*::mCherry reporter completely blocked endosome induction by CRY5B feeding (Fig. 5a). CRY5B exposure can be lethal in *C. elegans*, with most worms dying within 2 days [85]. We found that loss of *clp-4* significantly reduced the survival rate in worms exposed to CRY5B (Fig. 5b). Interestingly, both the *amph-1* deletion and the *unc-11* mutation also significantly decreased survival after CRY5B exposure, suggesting that dysregulation of the membrane repair process may generally sensitize *C. elegans* to this toxin.

### Induction of membrane repair increases tau phosphorylation in hippocampal neurons

**Fig. 6:**
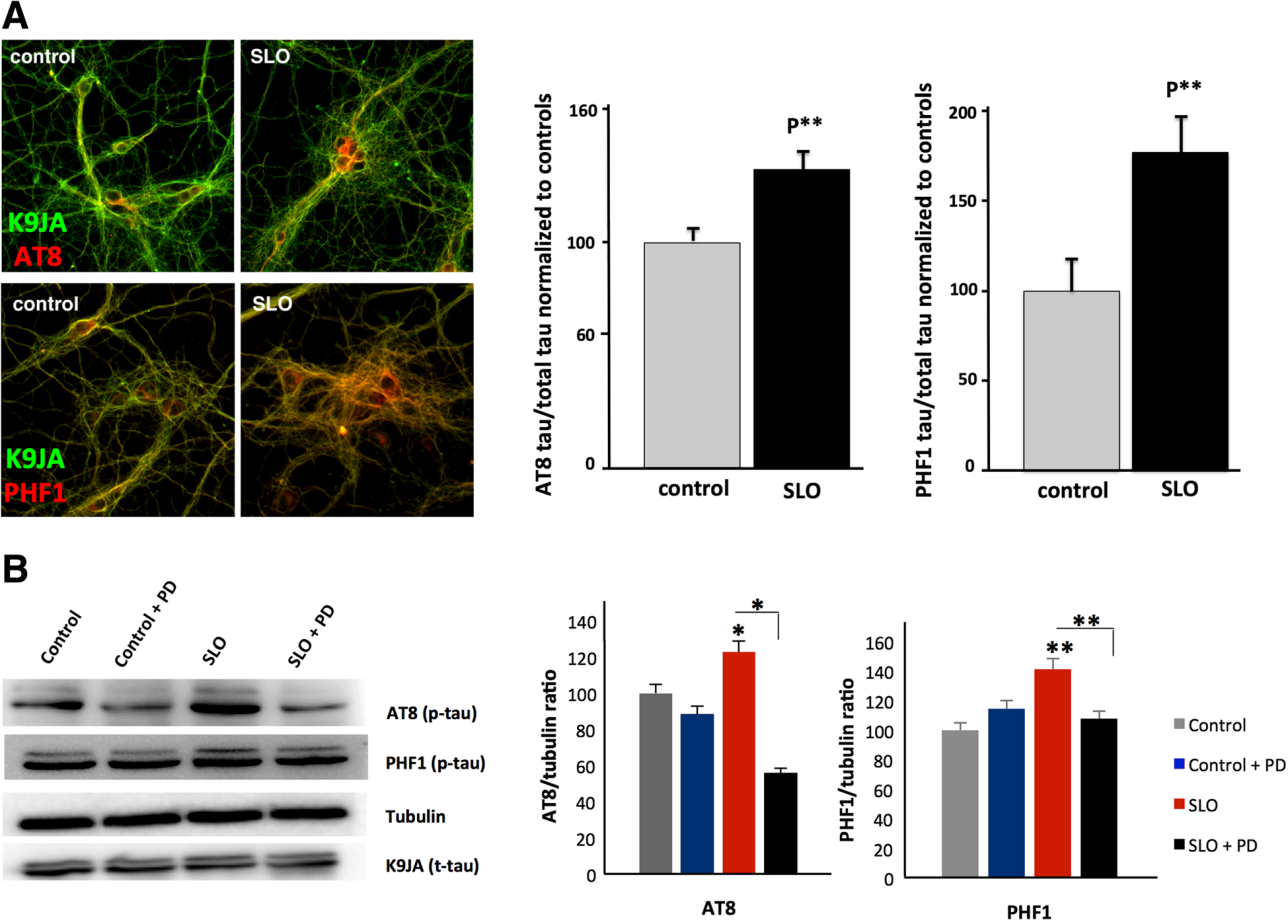
Treatment of hippocampal neurons with streptolysin O (SLO) increases tau phosphorylation. **a** Treatment of hippocampal neurons with SLO increases tau immunoreactivity at the AT8 and PHF1 phospho-epitopes, assayed by quantification of immunofluoresence images and normalized to total tau. **b** Treatment of hippocampal neurons with SLO increases tau immunoreactivity at the AT8 and PHF1 phospho-epitopes, assayed by quantification of immunoblots. *p < 0.05, **p < 0.01 and ***p < 0.001 when compared with the vehicle control

The *C. elegans* studies described above demonstrate that Aβ exposure can lead to a calpain-dependent induction of membrane repair. Multiple groups, including ours, have shown that hippocampal neurons exposed to Aβ oligomers show increased tau phosphorylation [14, 20, 76], and previous studies have also implicated calpain in this process [42, 62]. However, as *C. elegans* does not express any tau-like molecules in intestinal cells [21], we could not use the worm model to directly investigate the possibility that tau hyperphosphorylation is a downstream consequence of Aβ-induced membrane repair. We thus turned to cultured hippocampal neurons to test this hypothesis, which predicts that insults that induce membrane repair should also induce calpain-dependent tau phosphorylation. To induce membrane damage in hippocampal neurons we exposed them to streptolysin O (SLO), the bacterial pore-forming toxin used to establish the Andrews membrane-repair model. We observed that hippocampal neurons treated with 50 ng/ml SLO had increased tau phosphorylation at both the AT8 (Ser 202/Thr 205) and PHF1 (Ser 396/Ser 404) epitopes, assayed either by immunofluorescence (Fig. 6a) or immunoblot (Fig. 6b) (See Additional file 2, Individual Experiment Data). Critically, SLO induction of tau hyperphosphorylation was blocked by treatment with the calpain inhibitor PD150606 (Fig. 6b), demonstrating that tau hyperphosphorylation in response to a pore-forming toxin was calpain-dependent.

**Fig. 7:**
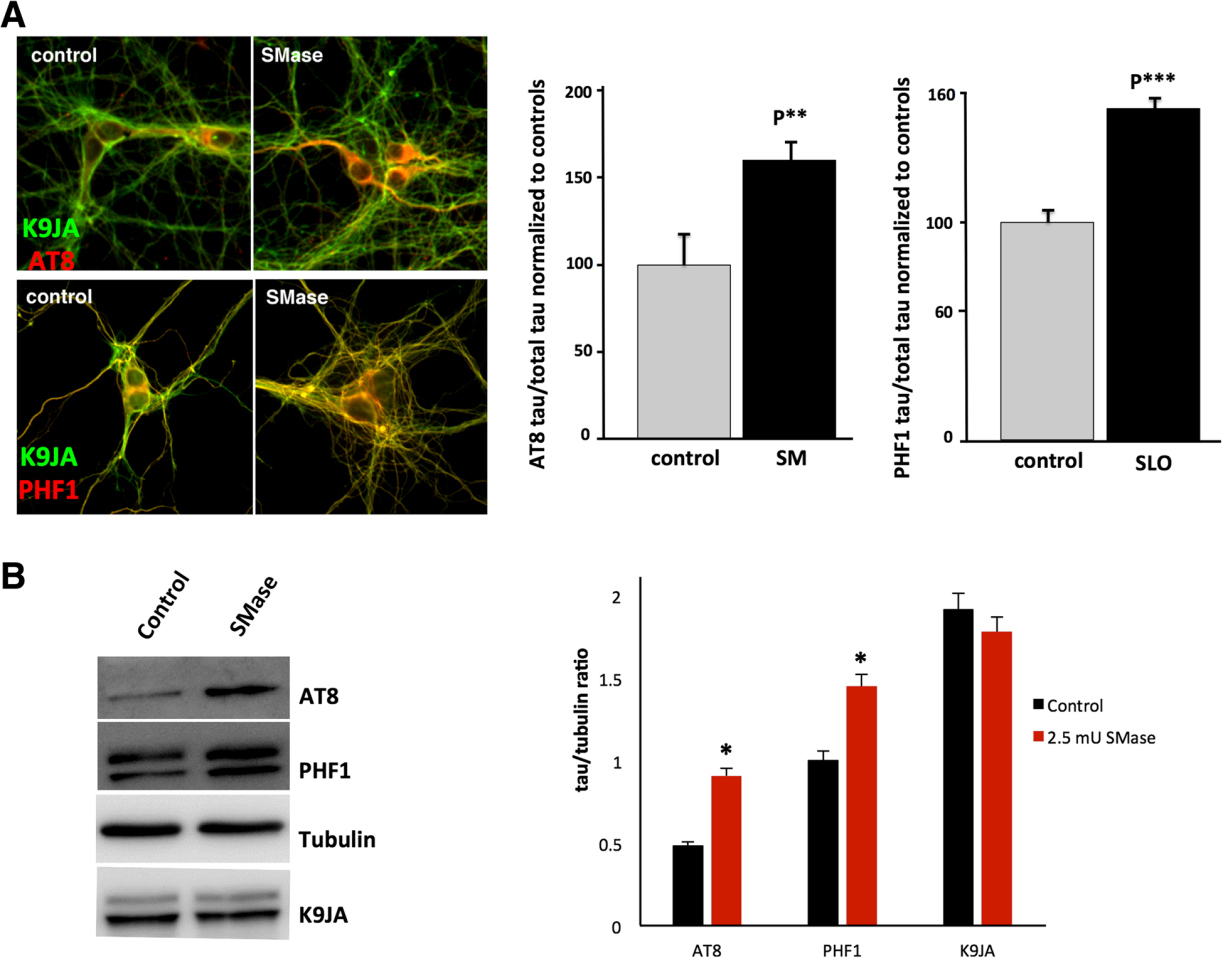
Treatment of hippocampal neurons with exogenous bacterial sphingomyelinase increases tau phosphorylation. **a** Cultured hippocampal neurons were treated with 2.5 mU/ml *B. cereus* sphingomyelinase, and tau immunoreactivity at the AT8 and PHF1 phospho-epitopes was quantified by immunofluorescence (normalized to total tau). **b** Cultured hippocampal neurons were treated with 2.5 mU *B. cereus* sphingomyelinase, and tau immunoreactivity at the AT8 and PHF1 phospho-epitopes was quantified by immunoblot (normalized to total tau). *p < 0.05, **p < 0.01 and ***p < 0.001 when compared with the vehicle control

As an independent test of the hypothesis that activation of membrane repair can lead to tau hyperphosphorylation, we sought to artificially stimulate this process by exposing hippocampal neurons to exogenous sphingomyelinase, a treatment that has previously been shown to promote the membrane repair process in HeLa cells [79]. We observed that hippocampal neurons treated with 2.5 mU *B. cereus* sphingomyelinase had increased tau phosphorylation at both the AT8 and PHF1 epitopes, assayed either by immunofluorescence (Fig. 7a) or immunoblot (Fig. 7b).

### Visualization of toxic Aβ oligomers using fluorescent biarsenical labeling

**Fig. 8:**
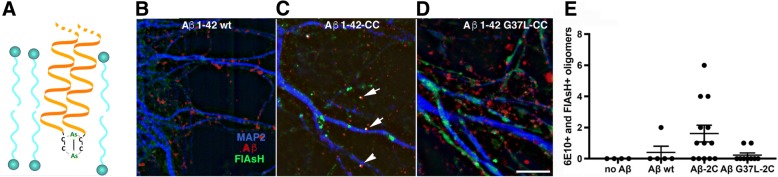
Biarsenical dye staining of dicysteine-tagged synthetic wild type and Gly^37^Leu variant Aβ_1–42_ in cultured hippocampal neurons. **a** Schematic model of how membrane-associated Aβ dimers in a parallel α-helical arrangement could bind the biarsenical FlAsH reagent. **b** Super-resolution image of cultured hippocampal neurons exposed to synthetic wild type Aβ_1–42_, treated with FlAsH reagent, fixed, permeabilized, and probed with anti-Aβ antibody 6E10. Note minimal association of FlAsH signal with Aβ immunoreactivity, expected because this synthetic peptide does not contain dicysteines. **c** Same experiment as described for panel “B”, except treatment with synthetic dicysteine-tagged wild type Aβ. Note multiple foci of co-localized FlAsH and Aβ staining (arrows). **d** Same experiment as described for panel “B”, except treatment with synthetic dicysteine-tagged Gly^37^Leu Aβ. Note this substitution prevents the formation of co-staining foci, supporting the hypothesis that the Gly^37^Leu substitution inhibits the assembly of (potentially pore-forming) multimers. (The increased neuronal process-associated FlAsH signal in the Gly^37^Leu Aβ -treated cultures may reflect increased non-specific uptake of the FlAsH dye, because neurons treated with the non-toxic Gly^37^Leu Aβ are healthier than neurons treated with wild type Aβ peptides.) **e** Quantification of co-labeling foci from multiple image fields acquired in the experiments described in **b-d**

The ability of Aβ to form ion-permeable pores in synthetic membranes is blocked by the Gly^37^Leu substitution, an observation that supports the “glycine zipper” model of Aβ multimerization [41]. We have previously shown that Aβ Gly^37^Leu oligomers cannot induce tau hyperphosphorylation in hippocampal neurons [20], and we show in the present study that this substitution prevents the induction of membrane repair in a *C. elegans* model. These observations are consistent with the idea that the Gly^37^Leu substitution interferes with the assembly of Aβ multimers capable of inducing membrane repair and subsequent downstream tau hyperphosphorylation, even though they do not directly demonstrate that this substitution interferes with Aβ multimerization per se. To demonstrate this directly, we sought a means to label multimeric Aβ specifically by taking advantage of the way that biarsenical compounds interact with appropriately positioned Cys residues. The ability of pairs of di-cysteine residues to coordinate the binding of biarsenical small molecules can be used to fluorescently label proteins tagged with a tetra-cysteine motif (FlAsH and ReAsH labeling, [1]). Subsequent studies demonstrated that di-cysteine residues located in different protein domains can also bind biarsenical fluorescent compounds if the di-cysteine residues are appropriately positioned in the protein [53]. The glycine zipper model predicts that α-helical interactions in a lipid environment will pair Aβ monomers such that their C-termini will be closely opposed, potentially allowing binding of biarsenical fluorescent probes (Fig. 8a).

We synthesized both wild type and Gly^37^Leu Aβ 1–42 tagged with two cysteine residues at the C-terminus. The addition of the C-terminal cysteines did not prevent wild type Aβ from inducing tau phosphorylation in hippocampal neurons (Additional file 1: Figure S3 A). Furthermore, *E. coli* engineered to express the two-Cys version of wild type, but not Gly^37^Leu, Aβ, induced endocytosis in the *C. elegans* model (Additional file 1: Figure S3B). We exposed hippocampal neurons to these Cys-tagged peptides using the same protocols we used previously to assay Aβ induction of tau. After 1 h exposure to Aβ oligomer preps, the cultured neurons were stained with 1:800 FlAsH dye for 30 min, then fixed, permeabilized and immunostained for Aβ (mAb 6E10) (see Methods). When imaged by super-resolution microscopy, neurons exposed to (untagged) Aβ 1–42 had foci of Aβ immuno-reactivity associated with neuronal processes, but as expected these rarely overlapped with the FlAsH reagent signal (Fig. 8b). In contrast, a number of Aβ-positive foci co-stained with the FlAsH reagent when the neurons were exposed to Cys-tagged Aβ 1–42 (Fig. 8c). Importantly, these co-staining-foci were reduced to the level of the untagged Aβ control in neurons exposed to Cys-tagged Aβ Gly^37^Leu (Fig. 8d, quantified in Fig. 8e). We interpret this result to indicate that the Gly^37^Leu substitution interferes with the Aβ multimerization required to interact with the FlAsH reagent.

## Discussion

We have developed a novel *C. elegans* model to investigate the cellular response to exogenous Aβ, and we observe an induction of endocytosis similar to that associated with membrane repair in response to exposure to the CRY5B pore-forming toxin [31, 38, 52, 85]. The observations that Aβ induction of endocytosis is blocked by a mutation in a gene encoding acid sphingomyelinase and the apparent membrane association of Aβ in induced endosomes also support the view that exogenous Aβ induces a membrane repair process as described in Fig. 1. To link membrane damage/repair with a classic marker of AD pathology, we also show that hippocampal neurons exposed to either a pore-forming toxin (streptolysin O) or exogenous sphingomyelinase display increased tau hyperphosphorylation comparable to that seen after exposure to Aβ. In addition, the increase in endosomes induced by either Aβ or CRY5B is inhibited by a loss-of-function mutation in *clp-4* and enhanced by mutations in *amph-1* or *unc-11*, which are orthologs of human LOAD genes BIN1 and PICALM, respectively. These results further tie the membrane damage/repair process to Alzheimer’s disease. Despite these similarities between the effects of Aβ-and CRY5B, feeding wild type worms Aβ-expressing *E. coli* does not result in the lethality observed in worms fed CRY5B. We attribute this difference to CRY5B being an evolved “professional” pore-forming toxin, assembled by a large protein that produces a pore size likely to be significantly larger [70] than a pore formed by Aβ oligomers. At present we cannot determine if the effects of Aβ in this model are due solely to the direct interaction of the peptide with the intestinal membrane, or whether proteins in the lumenal membrane are playing a role. We can conclude that suggested Aβ receptors (e.g., prion protein, NMDA glutamate receptors, α7nAChR) are highly unlikely to be involved, as there is no evidence these proteins are expressed in the *C. elegans* intestine.

The ability of the *clp-4* deletion to prevent endocytosis induced by Aβ or CRY5B implicates calpain activity in the membrane repair process. Calpains are known to target a number of cytoskeletal proteins [22], and to moderate cytoskeletal rearrangement, for example in focal adhesion remodeling [45]. Interestingly, in macrophages, calpain 2 cleavage of talin 1 is critical for endocytosis of the pore-forming “protective antigen” toxin produced by *Bacillus anthracis* [33]. We envision calpain activation as a conserved process required to induce local cytoskeletal changes that promote endocytosis in response to membrane damage.

The increased accumulation of Aβ-induced intestinal endosomes in the *amph-1* and *unc-11* mutants could result from either increased endosome formation or reduced endosome disassembly (or both). Loss of *amph-1* alters the distribution of the early endosome marker RAB-5 and leads to increases in the size of steady-state endosomes in the *C. elegans* intestine [49]. We note that an increase in the number and size of RAB-5 endosomes in AD brains has long been recognized [12]. BIN1, the ortholog of *amph-1*, has been implicated in the regulation of endocytosis in multiple contexts [65, 83, 86], even though its specific role in Alzheimer’s disease is unclear. The rs59335482 risk allele, a 3 bp insertion upstream of the BIN1 coding sequence, increases BIN1 transcription and is correlated with tau but not β-amyloid loads in AD brains [13]. This study also reported that loss of *Amph* (the Drosophila ortholog of BIN1) suppressed tau pathology, but not Aβ pathology, in transgenic fly models based on ectopic expression of these AD-associated proteins in the fly eye. However, studies in mammalian neurons have indicated that loss of BIN1 promoted the propagation of tau pathology [9]. Our studies cannot resolve the discrepancies between these reports, but they do establish that *amph-1* in *C. elegans* (and potentially BIN1 in mammals) play a role in the membrane repair response to pore-forming toxins.

*unc-11* encodes a *C. elegans* clathrin adaptor protein orthologous to AP180 and PICALM, two closely related mammalian proteins. The *unc-11* gene is expressed at high levels in neurons and at lower levels in other tissues, including the intestine. Interestingly, null alleles of *unc-11* do not prevent endocytosis of synaptic membrane, but do result in an increased size of synaptic endosomes [63]. Similarly, RNAi knockdown of PICALM in mammalian neurons leads to synaptic vesicles with variably increased size [69], suggesting that these proteins primarily regulate the sorting and recycling of endosomes rather than endocytosis per se. Thus, the increase of endosome number (and size) in the intestines of *unc-11* mutants exposed to CRY5B or Aβ likely results from altered endosome sorting or disassembly rather than upregulated endocytosis. Most studies support the view that PICALM expression is protective in AD, as full-length PICALM has been reported to be reduced in AD brain [3], and the protective allele of the primary AD-related SNP (rs3851179) is reported to increase PICALM mRNA levels in the brain [66].

The involvement of BIN1 and PICALM in endocytic processes has led multiple research groups to investigate their possible role in the production of Aβ, which largely occurs during the endocytic recycling of Amyloid Precursor Protein (APP) [56]. Indeed, loss of BIN1 has been reported to increase the production of Aβ [3, 60], whereas knockdown of PICALM is reported to reduce Aβ production [37, 80]. If, as described above, the risk alleles of BIN1 and PICALM lead to increased and decreased expression of these two genes, respectively, then the reported effects of these genes on Aβ production are actually the opposite of what might be expected. Our studies explore an alternative (but not mutually exclusive) explanation for the association of these genes with AD risk: a role in the cellular response to extracellular Aβ. We find that loss of function mutations in *amph-1* or *unc-11* sensitize worms to the CRY5B pore-forming toxin (Fig. 5b), and lead to similar dysregulation of the endocytosis induced by CRY5B or Aβ. These results are consistent with BIN1 and PICALM risk alleles acting by modulating cellular responses to extracellular Aβ.

The ability of *asm-1* mutations to suppress Aβ- or CRY5B-induced endocytosis supports the view that Aβ can instigate a membrane repair process analogous to that occurring in mammalian cells challenged by a pore-forming toxin such as SLO [32]. We reasoned that if Aβ acts as a pore-forming toxin in the AD brain, known effects of Aβ exposure such as tau hyperphosphorylation might be replicated using pore-forming toxins. We find that exposure of rat hippocampal neurons to SLO results in calpain-dependent tau hyperphosphorylation at two epitopes classically associated with AD pathology. Although we cannot exclude the possibility that SLO mimics Aβ-induced tau phosphorylation by an unrelated pathway, the ability of exogenous sphingomeylinase to induce tau hyperphosphorylation supports the view that tau hyperphosphorylation can be a downstream consequence of membrane damage/repair. Attempts to induce endocytosis in *C. elegans* intestines by feeding worms *B. cereus* sphingomeylinase were unsuccessful, possibly due to reduced activity of the enzyme at the lower temperature required for worm maintenance (20 °C for *C. elegans* vs. 37 °C for cultured neurons) and/or degradation of the enzyme in the intestinal lumen. Our experiments examining the effects of exogenous sphingomeylinase on tau hyperphophorylation also cannot determine if sphingomeylin-based secondary messengers are playing a role. Similarly, determining whether tau phosphorylation is a functional component of membrane repair or simply an incidental consequence will require additional investigation.

A major observation supporting the possible relevance of toxic Aβ pores is the dramatically reduced toxicity of the Aβ Gly^37^Leu variant in both transgenic *C. elegans* models and mammalian neurons [20]. This variant, which unlike wild type Aβ cannot induce ion-permeable channels in synthetic membranes, was investigated by the Bowie lab based on modeling studies that suggested it could not assemble pore-forming oligomers due to interference with a “glycine zipper” motif [41]. However, it had not been demonstrated previously that the Gly substitution in this critical variant actually alters Aβ multimerization, and in fact the Gly^37^Leu substitution does not reduce the stable oligomer species assayed by SDS-PAGE (see Additional file 1: Figure S1C). We therefore sought an approach to assay Aβ multimerization in vivo that could capture potentially less stable, membrane-associated oligomers. Using hippocampal neurons exposed to dicysteine-tagged synthetic Aβ and detection of closely associated pairs of dicysteine tags by means of a membrane-permeant biarsenical dye, we provide evidence that the Gly^37^Leu substitution does indeed inhibit Aβ multimerization in a cellular context. We suggest that the Aβ Gly^37^Leu variant can be used as a control peptide for investigating Aβ oligomer effects and could be superior to typical Aβ sequence-scrambled peptides used for this purpose.

More than 20 years ago, the discovery by Arispe and colleagues that Aβ could form ion channels in synthetic membranes led them to propose that Aβ membrane pores could contribute to Alzheimer pathology [6]. While multiple studies since have been supportive [36], this hypothesis has neither been directly supported (e.g., by the visualization of Aβ pores in AD pathological tissue), nor convincingly discounted. The failure to visualize Aβ pores in AD brains may not be surprising given the rapidity with which membrane pores are removed in mammalian cells (< 1 min in HEK293 cells; [32]), whereas the consequences of the repair process, such as hyperphosphorylation of tau, might accumulate. Our studies cannot directly prove the disease relevance of the pore-forming capacity of Aβ, but they do suggest a possible re-interpretation of existing human data. In particular, the demonstration of tau hyperphosphorylation as a downstream consequence of membrane damage/repair suggests mechanisms that may also be in play in non-AD tauopathies such as Chronic Traumatic Encephalopathy (CTE) and Niemann Pick type C disease [64]. Multiple explanations have been advanced to explain the dramatically increased AD risk for ApoE4 allele carriers (reviewed in [48]); our results support the idea that this risk could be due to altered membrane repair resulting from the reduced cholesterol and phospholipid secretion observed in APOE4 glia and neurons [54].

## Additional files

Additional file 1: Supplementary Figures. (DOCX 919 kb)

Additional file 2: Individual Experiment Data. (XLSX 21 kb)
