# Supplementary material for: In vivo induction of membrane damage by β-amyloid peptide oligomers
Source: Acta Neuropathol Commun. 2018 Nov 29;6:131. doi: 10.1186/s40478-018-0634-x (PMC6263551; doi:10.1186/s40478-018-0634-x)
Supplement: Supplementary file 1 — Supplementary Figures. (DOCX 919 kb) [file 40478_2018_634_MOESM1_ESM.docx]

**Additional file Figures.**


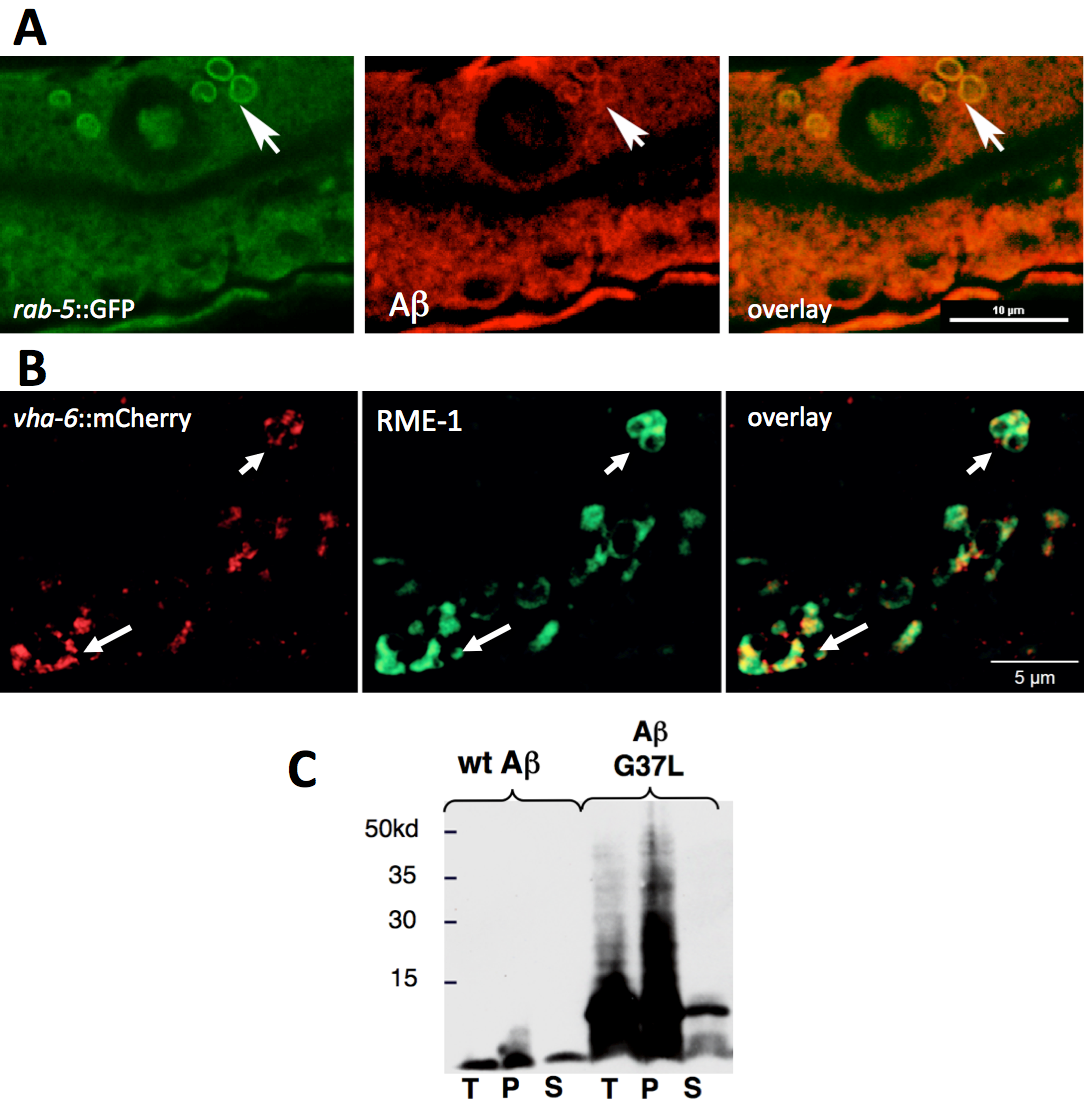


**Figure S1.** Intestinal vesicles induced by feeding *C. elegans* with *E. coli* expressing Aβ 1-42 express endosomal markers. **A.** Super resolution image of *rab-5*::GFP reporter strain (RT327) fed *E. coli* expressing Aβ 1-42, fixed, and probed with anti-GFP and anti-Aβ antibody (mAb 6E10). Note *rab-5*::GFP vesicles stain for Aβ (arrows). **B.** Super resolution image of intestinal endosomes induced in strain KWN117 by feeding *E. coli* expressing Aβ 1-42. Treated worms were fixed and probed with antibody against *C. elegans* endosomal marker protein RME-1. Note colocalization of *vha-6*::mCherry marker (relocalized from the intestinal lumen) and RME-1 (arrows). **C.** Anti-Aβ immunoblot (mAb 6E10) of *E. coli* cell lysates from engineered strains induced to express wild type or Gly^37^Leu variant Aβ 1-42 (equivalent culture inputs). Note that significantly higher levels of Aβ are expressed in the strain expressing the Gly^37^Leu variant, even though this strain fails to induce intestinal endocytosis when fed to *C. elegans*. (The difference in the amount of Aβ likely arises because wild type, but not Gly^37^Leu variant, Aβ impairs *E. coli* growth.) T = total, P = pellet, and S = supernatant.


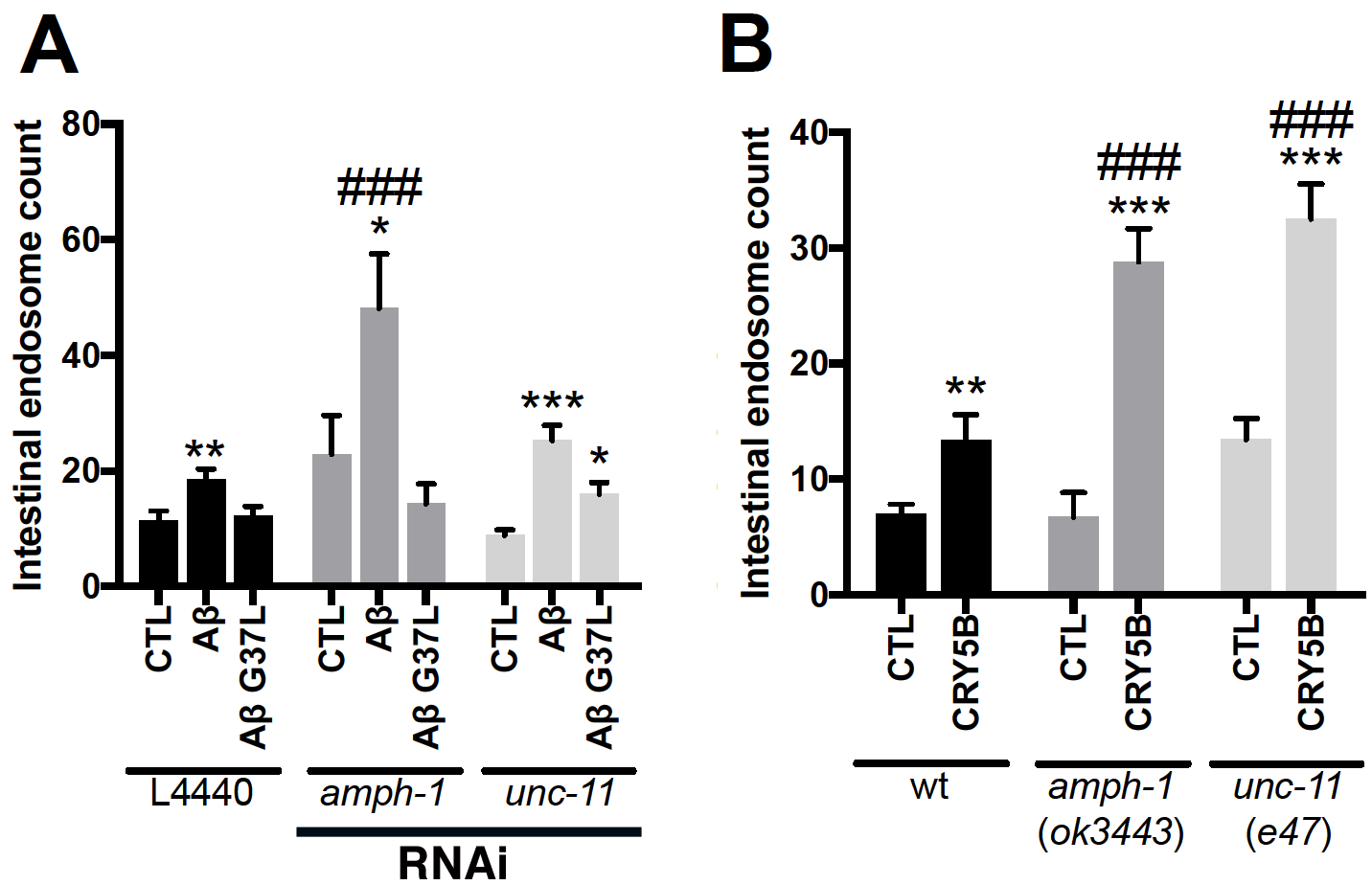


**Figure S2.** Effects of *amph-1* and *unc-11* loss of function on Aβ- and CRY5B-induced intestinal membrane damage.  **A.** RNAi knockdown of *amph-1 or unc-11* exacerbates Aβ-induced intestinal endosomes.  **B.** Deletion alleles of *amph-1* or *unc-11* exacerbate CRY5B-induced intestinal endosomes.  *p<0.05, **p<0.01 and ***p<0.001 when compared with the vehicle control and ###p<0.001 when compared with the wild type background or the L4440 empty control.


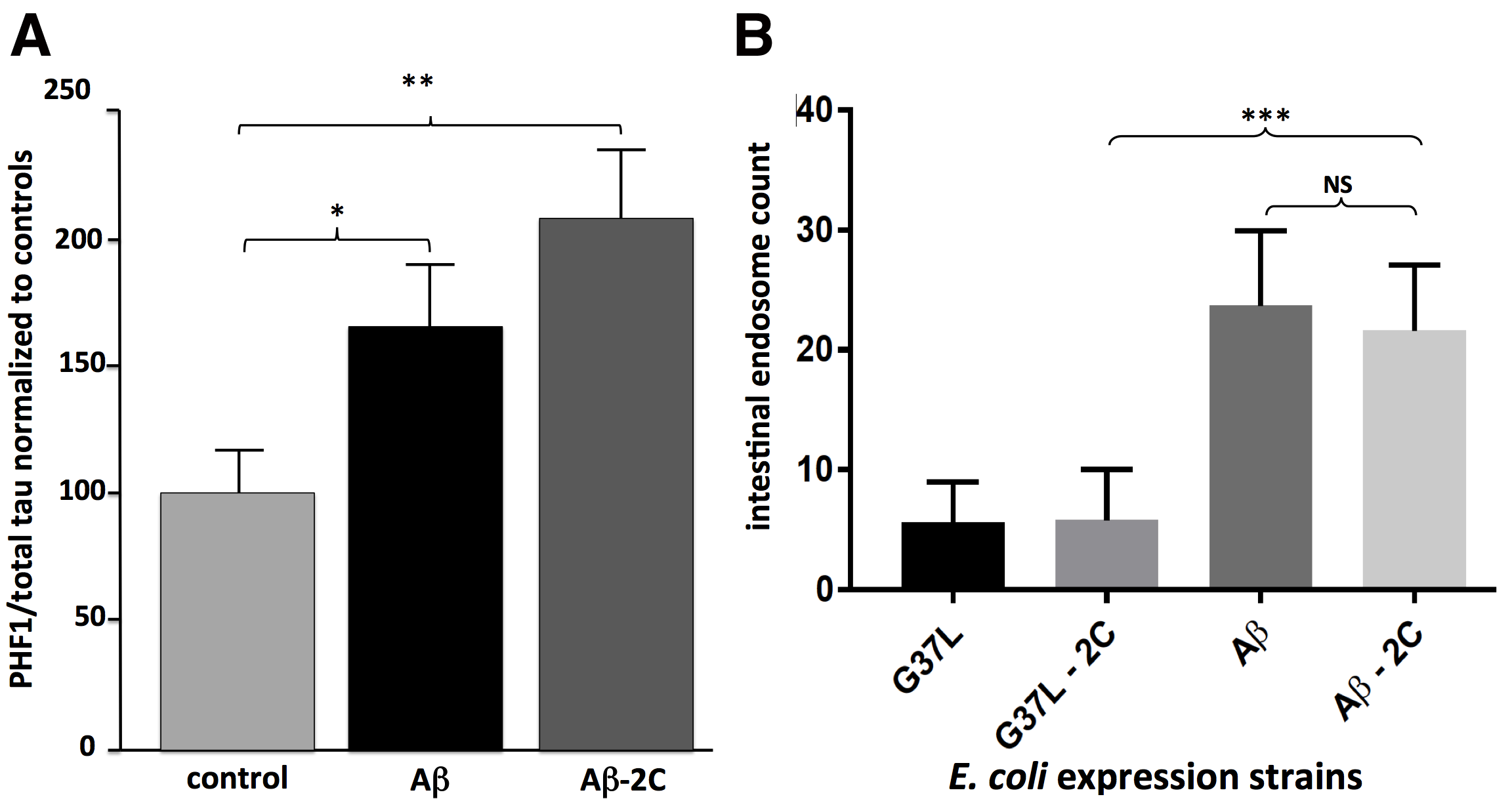


**Figure S3.** Cysteine-tagged Aβ induces tau hyperphosphorylation in hippocampal neurons and intestinal endosomes in *C. elegans*. **A.** Rat hippocampal neurons were exposed to 1 μM Aβ oligomers prepared from synthetic Aβ 1-42 with and without a terminal dicysteine tag, and levels of PHF1 and total tau reactivity were determined by quantitative immunofluorescence. Note the addition of cysteine residues to the Aβ peptide does not impair its ability to induce tau hyperphosphorylation. **B.** *C. elegans* reporter strain GK280 was fed *E. coli* induced to express wild type or Gly^37^Leu variant Aβ 1-42, with or without a dicysteine tag. Note that the addition of cysteine residues to the Aβ peptide does not alter the induction of intestinal endosomes.  *p<0.05, **p<0.01 when compared with the vehicle control, and ***p<0.001 comparing endosomes induced by *E. coli* expressing Aβ Gly^37^Leu or Aβ−2C Gly^37^Leu.
